# Role of the exposome in mental disorders: a scoping review protocol

**DOI:** 10.1136/bmjopen-2025-101575

**Published:** 2025-08-18

**Authors:** Claudia Gutierrez-Ortiz, Babul Hossain, Coralie Dessenne, Gloria A Aguayo, Maria Ruiz-Castell

**Affiliations:** 1 Socio-economic, Environmental Health & Health Services (CARES) Research Unit, Department of Precision Health, Luxembourg Institute of Health, 1A-B, rue Thomas Edison, L-1445 Strassen, Luxembourg; 2 Faculty of Science Technology and Medicine, University of Luxembourg, 2 Avenue de Université, L-4365 Esch-sur-Alzette, Luxembourg; 3 Science Office, Luxembourg Institute of Health, 1A-B, rue Thomas Edison, L-1445 Strassen, Luxembourg; 4 Deep Digital Phenotyping Research Unit, Department of Precision Health, Luxembourg Institute of Health, 1A-B, rue Thomas Edison, L-1445 Strassen, Luxembourg

**Keywords:** EPIDEMIOLOGY, MENTAL HEALTH, PSYCHIATRY, PUBLIC HEALTH, Review

## Abstract

**Abstract:**

**Introduction:**

The development of mental disorders is multifactorial across the lifespan. The introduction of the exposome concept has enhanced the understanding of life-course environmental factors by encompassing the totality of environmental exposures. While most studies on chronic diseases have applied a single-exposure approach, the exposome approach remains underutilised in mental disorder research. There is a need to better recognise the environmental factors considered in exposome analysis of mental disorders, the methodologies used and the gaps reported. This scoping review aims to map the evidence on the relationship between the exposome and mental disorders across the lifespan, identifying and describing the methodologies used and highlighting the gaps reported.

**Methods and analysis:**

This scoping review will follow PRISMA (Preferred Reporting Items for Systematic Reviews and Meta-Analyses) guidelines and the Population-Concept-Context approach. It will include observational and interventional studies involving populations of all ages in the community or healthcare settings. The search strategy will contain indexed terms in MEDLINE and will be adapted for CINAHL (EBSCO), Scopus, Embase and PsycINFO without restrictions on language or date of publication. For the selection of articles, two independent researchers will screen articles by title and abstract, followed by a full-text assessment. Afterwards, the extracted data will be summarised using a narrative and descriptive analysis.

**Ethics and dissemination:**

Ethics approval is not required for this scoping review. Dissemination activities will include peer-reviewed publications and academic presentations.

**STRENGTHS AND LIMITATIONS OF THIS STUDY**

- This study will follow the Joanna Briggs Institute’s recommendations for scoping review guidelines, ensuring clear and reliable results.
- Mental disorders encompass a large list of diseases, which may challenge reviewers due to the high number of citations and study timelines.
- Given the lack of consensus on the definition and analysis of the exposome, this scoping review faces the challenge of selecting which studies to include in the mapping, as many studies employ different approaches to the exposome.
- Studies with at least three exposures will be included, limiting the findings to research using a multi-exposure approach.

## Introduction

 The Diagnostic and Statistical Manual of Mental Disorders, Fifth Edition (DSM-5) defines mental disorder as a “syndrome characterised by clinically significant disturbance in an individual’s cognition, emotion regulation, or behaviour that reflects a dysfunction in the psychological, biological, or development processes underlying mental functioning”.^1^ It also classifies mental disorders into 20 diseases, such as schizophrenia, obsessive-compulsive disorders, depressive disorders, anxiety disorders, dissociative disorders and eating disorders (e.g., anorexia).^2^

To date, the Institute for Health Metrics and Evaluation has reported that the global prevalence of mental disorders ranges between 9.4% and 21.8%, with anxiety and depressive disorders being the most common. Furthermore, the prevalence of mental disorders increased by 48.1% between 2009 and 2019, reaching a total of 970.1 million cases.^3^ Although the incidence of mental disorders is expected to decrease over the next 20 years, the number of cases is projected to continue rising due to social factors and population growth.^4^

The aetiology of mental disorders is multifactorial across the lifespan, encompassing environmental and genetic factors.^5^ Initially, the scientific community focused on identifying diseases through the human genome using genome-wide association study^6^ or by adopting a ‘one-exposure-one-disease’ approach.^7^ Both approaches were limited: the former failed to explain much of the variability in diseases,^8^ while the latter did not account for the complexity of exposure patterns affecting diseases.^9^

In contrast, exposome analysis offers a holistic perspective. The term ‘exposome’ was introduced in 2005, broadening the understanding of how life-long environmental factors—such as urban conditions, lifestyle choices, social dynamics and chemical exposures starting from conception—contribute to disease. The exposome encompasses the totality of environmental exposures, their combinations and interactions.^9^,^11^

There are two exposomic approaches to identifying causal exposures: top-down (internal) and bottom-up (external).^12^ The top-down approach focuses on chemicals measured in biospecimens or blood samples. It can be classified into (1) internalised exogenous exposures (e.g., non-nutrient environmental molecules) and (2) endogenous nutrient exposures (e.g., gut microbiota). The bottom-up approach examines environmental chemicals (e.g., air, water, diet) and considers the combined effect of chemical, physical, psychological and social factors. This second approach can be classified into (1) general external exposures (e.g., built environment, climate) and (2) specific external exposures (e.g., chemical contaminants, diet).^13^ Integrating these four categories or combining these two approaches defines a functional exposome approach that links external exposure to internal dose, biological response and diseases.^13^

Although exposome analysis offers a holistic perspective, it faces several challenges, including the high dimensionality of exposome data, the combined effects of exposures, the integration of data omics and the evaluation of causality. While methods to address these challenges have been published, additional information—particularly from longitudinal exposome datasets—is still needed.^9^ Future interventions must differentiate the measured components from the various sources of exposure.^13^

The role of the exposome in non-communicable diseases has been described in a previous scoping review. The authors concluded that much remains to be explored in mental disorders.^14^ Recent studies have assessed mental disorders under an exposome-wide association study (ExWAS) approach. For example, a cross-sectional study investigated the association between environmental toxicants and depressive symptoms by analysing multiple volatile organic compounds.^15^ Also, two cohort studies have explored the role of lifestyle and environmental factors on suicide attempts^16^ and the impact of the digital exposome on youth mental health,^17^ developing exposome scores for each outcome.

The studies mentioned above highlight the need for a deeper understanding of how exposome research has been conducted so far and the identification of key characteristics in exposome analysis related to mental disorders. Due to the high dimensionality, diversity of exposure data and the increased use of the exposome approach, a scoping review will be performed. A preliminary search of MEDLINE, the Cochrane Database of Systematic Reviews and *Joanna Briggs Institute (JBI) Evidence Synthesis* revealed no current or underway systematic or scoping reviews on the topic. Therefore, we want to fill this gap by performing a scoping review.

This scoping review aims to map the evidence on the relationship between the exposome and mental disorders in all populations from birth to older ages, identify and describe the methodologies used, and highlight the gaps reported in the literature.

## Methods and analysis

A draft version of this scoping review was registered on the Open Science Framework and will be conducted following the JBI methodology for scoping reviews.^18^ The research questions were determined based on the objectives and with a previous consensus among all authors in February 2025. The final manuscript is expected to be completed between October and November 2025.

### Eligibility criteria

The inclusion criteria will follow the Population-Concept-Context framework.^18^ Their definitions are described in detail in table 1. Briefly, the population will include all genders and ages; the concept will consist of studies examining the association between the exposome and mental health, and the context will consist of the general population and clinical settings. These criteria will support the study selection and data extraction based on the objectives and research questions.^19^

**Table 1:** Eligibility criteria

Objective	Research question	Population	Concept	Context
To map the evidence on the relationship between exposome and mental disorders.	How is the exposome defined in studies examining the relationship between environmental exposures to mental disorders in the general population, both in community and healthcare settings?	All populations, including adults, pregnant women and children.	Definition of exposome in studies linking environmental exposures to mental disorders.	Studies conducted in community or healthcare settings without restrictions on cultural factors, geographical location, ethnicity or gender.
	Which environmental factors are associated with mental disorders under an exposome approach in the general population in the community or healthcare settings?		Identification of the environmental factors (at least 3) associated with mental disorders under an exposome approach.	
To identify and describe the methodologies applied in original studies that examine the relationship between the exposome and mental disorders.	What methodologies are used to analyse the relationship between the exposome and mental disorders in the general population in the community or healthcare setting?	All populations, including adults, pregnant women and children.	The methods applied in studies of mental disorders with an exposome analytical approach.	Studies conducted in community or healthcare settings without restrictions on cultural factors, geographical location, ethnicity or gender.
To highlight gaps reported in original studies that examine the relationship between the exposome and mental disorders.	What research gaps exist in exposome studies examining the relationship between environmental exposures and mental disorders in the general population in the community or healthcare setting?	All populations, including adults, pregnant women and children.	Research gaps reported in studies of mental disorders with an exposome analytical approach.	Studies conducted in community or healthcare settings without restrictions on cultural factors, geographical location, ethnicity or gender.

This scoping review will use a bottom-up approach. Potential sources or methods applied in the studies could be geospatial methods, census data (neighbourhood characteristics), public databases, personal air samplers (e.g., wearable devices), social media platforms, etc.^13^

The general characteristics of an exposome analysis are assessment across multiple exposure domains, integration of data across various scales of variation (e.g., populations, over time, and space) and high dimensionality of exposure data.^20^ The definition of exposome implies a broad and holistic set of exposures across an individual’s lifetime. One of our objectives is to map novel agnostic and data-driven methodologies used to characterise the exposome and its effects on mental disorders.^21^ However, there is currently no consensus on the minimum number of variables required to perform those exposome analyses, which has been identified as a key challenge in the field.^11^ We propose including at least three exposure variables in our studies to ensure the application of such methodologies and avoid including studies that do not follow this approach, such as single-exposure models or two-variable interactions, which have already been extensively documented in the literature.^21^,^23^

### Type of evidence

The types of sources of this scoping review will focus on peer-reviewed and original human studies without language restriction. These include observational studies (prospective and retrospective cohort studies, case-control studies and cross-sectional studies) and interventional studies in community or healthcare settings.

### Exclusion criteria

Studies will be excluded if they are not original research (e.g., reviews, systematic reviews, meta-analyses), case reports, qualitative research, pilot studies, guidelines or protocols, preprints, books, position statements, conference abstracts or if the full text is not available.

### Search strategy

The search strategy will follow a three-step process. First, an initial search of MEDLINE via PubMed to identify relevant articles on the topic. We will identify index terms and keywords to guide the research, followed by a screening using ‘Medical Subject Headings’ (MeSH) or ‘text word’ (TW) as specific fields. The search strategy is described in box 1. Second, the search strategy will be adapted for other databases, such as CINAHL (EBSCO), Scopus, Embase and PsycINFO, without restrictions on language or date of publication. An expert librarian (CD) will validate the strategy. Third, the reference list of the included studies in the systematic reviews or meta-analyses on the same topic will be screened for additional relevant studies (snowballing).^24^

Box 1 **Search strategy in MEDLINE (PubMed) conducted on 4 February 2025**

#1: “Mental Disorders” [(MeSH])

#2: “Exposome” [(MeSH])

#3: “Exposome” [(MeSH]) OR Exposom*[(TW])

#4: “Environmental Exposure” [(MeSH]) OR “Environmental Exposure*” [(TW])

#5 : #1 AND (#3 OR #4)

MEDLINE, Medical Literature Analysis and Retrieval System Online; MeSH, Medical Subject Headings; TW, text words.

### Study selection

The selection of articles will be using CADIMA V.2.2.4.2/2023. This client-server software application will support the key steps of this scoping review, such as management of search results and removal of duplicates, guidance during the selection process with a performance of consistency check, management and conduct of data extraction, and management of the critical appraisal process.^25^

Following the search, all identified citations will be collected and uploaded to CADIMA, and duplicates will be removed. A sample of 50 articles will be randomly selected to perform a pilot test to ensure consistency of screening among reviewers. Next, two independent reviewers (CG-O, BH) will screen by title and abstracts, considering the inclusion and exclusion criteria. Afterwards, the selected citations will be evaluated in full text, considering the same criteria by the two independent reviewers (CG-O, BH). Excluded articles and the reasons for their exclusion will be reported. Any disagreements between the two independent reviewers will be resolved by consulting a third reviewer (MR-C or GAA). The search and study inclusion process results will be fully reported in the final scoping review and presented in a PRISMA-Scr (Preferred Reporting Items for Systematic Reviews and Meta-Analyses extension for scoping reviews) flow diagram explained elsewhere.^18^

### Data extraction

Two independent reviewers (CG-O and BH) will extract data from papers included in the scoping review using a data extraction form based on the JBI manual.^18^ A draft data extraction form is provided in table 2. It will include specific details about the participants, concept and context.

**Table 2:** Data extraction form

General information of study			
Number of study		1	2
Title			
Author’s name			
Year of publication			
Country			
Study design			
Objectives			
**Population-Concept-Context data**			
Population	Number of participants		
	Mean age		
	Sex (% of females and males)		
	Ethnicity		
	Specific conditions (pregnancy, offspring, etc)		
Concept	Definition of the exposome		
	Types of domains		
	Number of exposures		
	Types of exposures		
	Sources of exposure data		
	Time of exposure (months or years)		
	Duration of follow-up (mean)		
	Type of mental disorders		
	Psychiatric phenotype reported		
	Time of diagnosis (years)		
	Main findings		
	Gaps reported		
	Limitations		
Context	Community		
	Healthcare settings		

Reviewers may modify and revise the data extraction form as needed during the pilot phase of the process. Any disagreements will be resolved through discussion. If additional information is missing, the authors will be contacted to provide it.

### Critical appraisal of available literature

A quality assessment will be done in the included studies. We will use the RoB (Risk of bias) 2.0 tool for interventional studies, whereas, in observational studies, we will use the Newcastle-Ottawa adapted for cohort and case-control studies and the JBI tool for cross-sectional studies.^26^

### Data analysis and presentation

Results will be presented with descriptive statistics (e.g., frequency analysis and percentages) using tables and diagrammatic forms. In addition, we will synthesise the information and highlight key extracted data into conceptual categories, which will correspond to the objectives and research questions.^18^ A narrative description will be used to provide detailed information to the reader.

### Ethics and dissemination

Due to the study design, ethics approval is not applicable. The dissemination will be through peer-reviewed publications and academic presentations at conferences.
