# Supplementary material for: Role of the exposome in mental disorders: a scoping review protocol
Source: BMJ Open. 2025 Aug 18;15(8):e101575. doi: 10.1136/bmjopen-2025-101575 (PMC12366602; doi:10.1136/bmjopen-2025-101575)
Supplement: Supplementary file 1 [file bmjopen-2025-101575.reviewer_comments.pdf]

PEER REVIEW HISTORY

BMJ Open publishes all reviews undertaken for accepted manuscripts. Reviewers are asked to complete a checklist review form and are provided with free text boxes to elaborate on their assessment. These free text comments are reproduced below.

ARTICLE DETAILS

Title (Provisional)

The role of the exposome in mental disorders: a scoping review protocol

Authors

Gutierrez-Ortiz, Claudia; HOSSAIN, BABUL; Dessenne, Coralie; Aguayo, Gloria A.; Ruiz-Castell, Maria

---

VERSION 1 - REVIEW

**Reviewer**	**1**
**Name**	**Guloksuz, Sinan**
**Affiliation**	**Maastricht University**
**Date**	**09-May-2025**
**COI**	**None**

This is a timely and well-conceived scoping review protocol that aims to systematically explore the application of the exposome paradigm in mental health research. The proposal is well-written, the methodology is rigorous, and it conforms to the PRISMA guideline. The topic addresses a major gap in psychiatric epidemiology by aiming to synthesize how the multiplicity of environmental exposures has been investigated in the context of mental disorders. I have only a few suggestions/questions that could further improve the manuscript.

The authors propose using (at least 3 environmental factors) as the threshold for defining the exposome, but this cutoff seems somewhat arbitrary and may exclude studies focusing on two interacting exposures. It would be beneficial to thoroughly justify this criterion and discuss its limitations.

In terms of psychiatric phenotypes, will subclinical phenotypes and dimensional psychopathology be included? I firmly believe that this is essential given the transdiagnostic nature of psychopathology.

There are a few typos (line 26: While l studies… in the abstract).

**Reviewer**	**2**

**Name**	**van Kamp, Irene**
**Affiliation**	**RIVM, cMGO**
**Date**	**25-Jun-2025**
**COI**	**None**

The protocol looks fine and adequate to me. My point of doubt concerns the possibility of too few studies to perform a proper systematic review, but also the heterogeneity of the exposome/total exposure defintion. From recent experience I know that the number of eligible papers will be extremely limited. I miss reference to the recent reviews performed already into the topic (in the protocol it states:........revealed no current or underway systematic or scoping reviews on the topic Therefore, we want to fill this gap by performing a scoping review)... this is not correct. .....explanations of alternatives to deal with this eg turning back to single exposure papers to built up the evidence. I think it would be good to add a strategy in case this problem is encountered as well. This could be done under study limitations..... also I would suggest to critically look into the definition of exposome, merely counting the number of exposures included in the study does not suffice, and the odds are small that the studies are comparable..... standing in the way of a proper meta-analysis. I am aware that this is recognised in the statement: Given the lack of scientific consensus on the exposome definition and

analysis, this study faces the challenge of selecting and summarising

heterogeneous evidence from different exposome concepts. Nevertheless,

the reviewers will pilot a sample of articles to reach an agreement on the

eligibility criteria >>> I would expand on that ....

VERSION 1 - AUTHOR RESPONSE

FIRST REVIEWER

1. Comments

This is a timely and well-conceived scoping review protocol that aims to systematically explore the application of the exposome paradigm in mental health research. The proposal is well-written, the methodology is rigorous, and it conforms to the PRISMA guideline. The topic addresses a major gap in psychiatric epidemiology by aiming to synthesize how the multiplicity of environmental exposures has been investigated in the context of mental disorders. I have only a few suggestions/questions that could further improve the manuscript.

The authors propose using (at least 3 environmental factors) as the threshold for defining the exposome, but this cutoff seems somewhat arbitrary and may exclude studies focusing on two interacting exposures. It would be beneficial to thoroughly justify this criterion and discuss its limitations.

Author's answer:

Thank you for your comment. There is no consensus regarding the minimum number of variables required to study the impact of the exposome on health. One of our objectives was to analyse the novel agnostic and data-driven methodologies used to characterize the exposome and its effects on mental disorders, methods that typically integrate data from different methodologies and time points (references). To avoid including studies that do not follow this approach, we propose a minimum of three exposure variables. We have updated our sentence (page 7-8, lines 144-153) *"The definition of the exposome implies a broad and holistic set of exposures across an individual's lifetime. One of our objectives is to map novel agnostic and data-driven methodologies used to characterize the exposome and its effects on mental disorders (references). However, there is currently no consensus on the minimum number of variables required to perform those exposome analysis, which has been identified as a key challenge in the field (reference). We propose including at least three exposure variables in our studies to ensure the application of such methodologies and avoid including studies that do not follow this approach, such as single-exposure models or two-variable interactions, which have already been extensively documented in the literature. "*

We have also added this information in the limitation section (page 3, lines 54-55) "*Studies with at least three exposures will be included , limiting the findings to research using a multi-exposure approach."*

2. Comments

In terms of psychiatric phenotypes, will subclinical phenotypes and dimensional psychopathology be included? I firmly believe that this is essential given the transdiagnostic nature of psychopathology.

Author's answer:

Thank you for this suggestion. We agree that the subclinical phenotypes and dimensional psychopathology should be included and we will follow your recommendation. Although the keywords used in our search strategy are based on the classification from DSM-5, the field terms across all databases ensure the retrieval and inclusion of studies addressing subclinical phenotypes and dimensional psychopathology.

3. Comments

There are a few typos (line 26: While l studies… in the abstract).

Author's answer:

Thank you for this precision. The sentence has been corrected. The text reads: *"While most studies on chronic diseases have applied a single-exposure approach, the exposome approach remains underutilized in mental disorder research".*

SECOND REVIEWER

1. Comments

The protocol looks fine and adequate to me. My point of doubt concerns the possibility of too few studies to perform a proper systematic review, but also the heterogeneity of the exposome/total exposure defintion. From recent experience I know that the number of eligible papers will be extremely limited.

Author's answer:

Thank you for this comment. We agree on the heterogeneity of the exposome definition. Therefore, we are conducting a scoping review with a broad and exploratory approach, rather than a systematic review or meta-analysis with a focused approach, aiming for more comprehensive results. Due to concerns about a limited number of publications, we have performed an exploratory search (date 08-07-25) in Medline using the strategy described in Table 2 of the main text. This search yielded a

total of 5,955 publications using the following strategy: ((“Environmental Exposure”[Mesh]) OR “Exposome”[Mesh]) AND “Mental Disorders”[Mesh]. This number does not yet reflect the final number of studies that we will include, as we will also consider four additional databases: CINAHL (EBSCO), Scopus, Embase, and PsycINFO to ensure a sufficient number of studies for inclusion.

2. Comments

I miss reference to the recent reviews performed already into the topic (in the protocol it states:........revealed no current or underway systematic or scoping reviews on the topic Therefore, we want to fill this gap by performing a scoping review)... this is not correct. .....explanations of alternatives to deal with this eg turning back to single exposure papers to built up the evidence. I think it would be good to add a strategy in case this problem is encountered as well. This could be done under study limitations.....

Author’s answer:

Before submitting this manuscript and the current point-by-point answer, we were unable to find a scoping review on the exposome approach for mental disorders. We did a search in Medline and Scopus (date: 08-07-2025) using the followed strategy: For Medline, ((scoping review[Title/Abstract]) AND (exposome[Title/Abstract])) AND (mental[Title/Abstract]), and for scopus ("Exposome" AND 'scoping review' AND "mental").

While there are existing literature reviews on the exposome and health, few studies focus on the role of the exposome on mental disorders, and when they do, they do not adopt a scoping review approach. For instance, Haddad et al. (2019) (https://doi-org.proxy.bnl.lu/10.1007/s40726-019-00130-7) conducted a scoping review on the exposome and health, but it did not address mental disorders. Other publications, such as those by Erzin et al. (2021) (doi: 10.5152/alphapsychiatry.2021.21307) and Guloksuz et al. (2018) (doi: 10.1001/jamapsychiatry.2018.1211), are not scoping reviews.

3. Comments

also I would suggest to critically look into the definition of exposome, merely counting the number of exposures included in the study does not suffice, and the odds are small that the studies are comparable..... standing in the way of a proper meta-analysis. I am aware that this is recognised in the statement: Given the lack of scientific consensus on the exposome definition and analysis, this study faces the challenge of selecting and summarising heterogeneous evidence from different exposome concepts. Nevertheless, the reviewers will pilot a sample of articles to reach an agreement on the eligibility criteria >>> I would expand on that ....

Author’s answer:

Thank you for this suggestion. We agree that the terms used in the limitation section might be confusing. We have revised it to clarify that we are conducting a scoping review rather than a meta-analysis (please, see page 3, lines 51-53). Furthermore, the results of this scoping review may guide future systematic review and meta-analysis. Now the text reads:
“Given the lack of scientific consensus on the definition and analysis of the exposome, this scoping review faces the challenge of selecting which studies to include in the mapping, as many studies employ different approaches to the exposome.”

Furthermore, we have removed the last sentence *“Nevertheless, the reviewers will pilot a sample of articles to reach an agreement on the eligibility criteria”* from the limitation section in response to

the editor’s comments about sentence length. However, a more detailed explanation is included in the study selection section (page 12, lines 178–193).
